# Theoretical Study of the Interaction between Graphitic Carbon Nitride and 2,6-Dichloro-1,4-benzoquinone Pollutants: Environmental Applications

**DOI:** 10.1021/acsomega.5c12928

**Published:** 2026-05-18

**Authors:** Sara Ruth Ramos Rocha, Nailton Martins Rodrigues, Michael González-Durruthy, Silvete Guerini

**Affiliations:** † Laboratório de Simulação, 37892 Universidade Federal do Maranhão, 65080-805 São Luís, MA, Brasil; ‡ Laboratório de Química Computacional, Universidade Federal do Maranhão, 65080-805 São Luís, MA, Brasil; § LAQV@REQUIMTE/Department of Chemistry and Biochemistry, Faculty of Sciences, University of Porto, 4169-007 Porto, Portugal

## Abstract

Halobenzoquinones (HBQs) are an emerging class of disinfection byproducts of growing environmental concern due to their high toxicity and persistence in aquatic systems. In this work, we investigate the adsorption behavior of 2,6-dichloro-1,4-benzoquinone (DCBQ) on two phases of graphitic carbon nitride—triazine-based (t-g-C_3_N_4_) and tri-s-triazine-based (h-g-C_3_N_4_)—combining Grand Canonical Monte Carlo (GCMC) simulations with first-principles density functional theory (DFT) calculations. GCMC was employed to identify the most probable adsorption configurations and competitive water coadsorption under realistic aqueous conditions. The results reveal that h-g-C_3_N_4_ exhibits a significantly higher adsorption capacity and stronger affinity toward DCBQ compared to t-g-C_3_N_4_, even at high water ratios, highlighting its superior selectivity for pollutant capture. DFT calculations were then applied to refine the structural, energetic, and electronic properties of the selected configurations. Both g-C_3_N_4_ phases exhibit physisorption-driven interactions with DCBQ, accompanied by band gap modulation and the emergence of molecular states near the Fermi level. Noncovalent interaction (NCI) and reduced density gradient (RDG) analyses further demonstrate that adsorption is governed primarily by dispersion forces and π–π interactions. Additionally, isolated dimer calculations show that DCBQ molecules can stabilize one another through favorable noncovalent contacts, supporting the cooperative adsorption behavior observed in GCMC simulations. Overall, our results provide a comprehensive multiscale description of DCBQ adsorption on g-C_3_N_4_ and reveal that h-g-C_3_N_4_ is a promising candidate material for the selective capture of halobenzoquinones in aqueous media. This study advances the understanding of pollutant–surface interactions and contributes to the development of nanostructured adsorbents for water purification applications.

## Graphical abstract

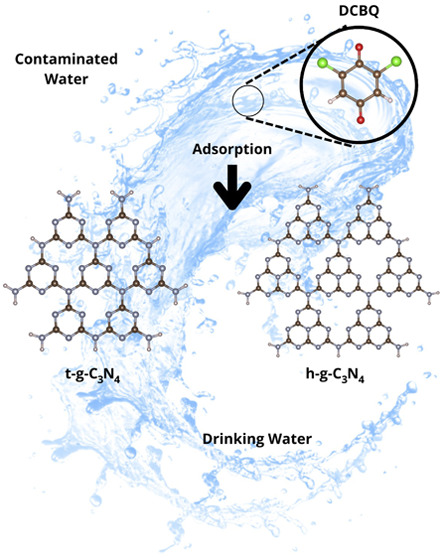

## 1 Introduction

Water treatment is essential to ensure public quality water, eliminating pathogenic microorganisms and harmful chemical substances. However, the use of chemical agents, such as chlorine and chloramine, can lead to the formation of unwanted byproducts, the disinfection byproducts (DBPs), resulting from the interaction between disinfectants and natural organic matter (NOM) present in the water.^1,2^ Epidemiological studies indicate that prolonged exposure to these compounds, particularly through the consumption of drinking water, is associated with adverse health effects, including an increased risk of bladder cancer.^3^ Among the main DBPs, trihalomethanes (THMs) and haloacetic acids (HAAs) stand out, as they are regulated due to their known toxicity.^4^ However, an emerging group of DBPs, the halobenzoquinones (HBQs), has raised growing concern due to their toxic potential and significant presence in treated water.^3,5^

HBQs represent a highly reactive class of DBPs formed by the interaction of chemical disinfectants with natural organic matter or organic compounds introduced into the process, such as residues from personal care products. Studies indicate that HBQs exhibit significantly higher toxicity compared to other regulated DBPs, as they can induce the formation of reactive oxygen species (ROS), leading to oxidative stress, DNA damage, and cellular alterations.^6,7^ Additionally, their ability to form adducts with DNA increases the risk of genetic mutations and is directly related to carcinogenicity, particularly in tissues such as the bladder.^3,8^ Among these substances, 2,6-dichloro-1,4-benzoquinone (DCBQ) stands out due to its high abundance in treated water, with higher concentrations observed in systems using free chlorine for disinfection.^9^

Given this scenario, it is essential to develop effective strategies to identify and remove these toxic substances from treated water. In recent years, advanced materials integrating light-harvesting and catalytic functionalities have emerged as promising platforms for wastewater remediation. For instance, ternary organic photovoltaic films have been successfully applied to wastewater treatment by promoting fast exciton separation and efficient free radical generation, achieving rapid removal of heavy metal ions and organic contaminants under solar irradiation.^10^ Such strategies highlight the importance of electronic structure modulation, charge separation efficiency, and reactive species generation in environmental remediation technologies. These advances reinforce the need to explore materials with tunable electronic properties, not only for efficient pollutant removal but also for applications where adsorption-induced electronic perturbations may enable sensing functionalities. A promising alternative is the use of nanostructured materials, such as Graphitic Carbon Nitride (g-C_3_N_4_). This two-dimensional material, composed of π-conjugated triazine and tri-s-triazine units interconnected by tertiary amine groups, exhibits high thermal and chemical stability, in addition to being nontoxic and low-cost.^11^ Its unique structure and photochemical properties make g-C_3_N_4_ an attractive candidate for environmental applications,^12^ offering a sustainable approach for the removal of emerging contaminants, such as HBQs, from drinking water. In particular, g-C_3_N_4_ has demonstrated excellent photocatalytic activity under visible light for the degradation of a wide range of organic pollutants in aqueous media—including pharmaceuticals, dyes, pesticides, phenols, and phenolic compounds—through the generation of reactive oxygen species.^13,14^ Qi et al. provided a comprehensive review on the environmental applications of g-C_3_N_4_, highlighting its effectiveness in degrading various pollutants such as phenol, bisphenol A, nitrophenols, dyes (e.g., methylene blue, rhodamine B), antibiotics, pesticides, and aniline.

The photocatalytic mechanism involves the generation of reactive oxygen species (ROS), including hydroxyl radicals (OH), superoxide radicals (O_2_^–^), and hydrogen peroxide (H_2_O_2_), resulting from visible-light-induced excitation and charge separation in the g-C_3_N_4_ structure.^13^ In a complementary study, Warshagha and Muneer synthesized phenyl-modified graphitic carbon nitride (Ph-g-C_3_N_4_) via a one-step calcination process. The modified materials exhibited enhanced photocatalytic performance for the degradation of an azo dye (methyl orange) and a pharmaceutical derivative in aqueous solution under visible light. The improvements were attributed to the incorporation of phenyl groups, which increased light absorption, reduced the band gap energy, improved the surface area and porosity, and promoted effective charge separation. Quenching experiments confirmed that ROS, particularly superoxide and hydroxyl radicals, played a major role in the photocatalytic degradation process.^13^ Furthermore, various modification strategies such as doping and heterojunction formation have been shown to significantly enhance its efficiency, reinforcing its potential as an advanced material for water purification technologies.^15–17^

From a theoretical perspective, first-principles methods such as Density Functional Theory (DFT) have proven highly effective for describing adsorption processes and charge transfer in two-dimensional systems. Building on this, several studies have highlighted the potential of g-C_3_N_4_ as an adsorbent for environmental applications. Luo et al. demonstrated that Pt doping significantly enhances the interaction of g-C_3_N_4_ with H_2_ molecules, where DFT calculations revealed stronger adsorption energetics, band gap narrowing, and noticeable charge transfer effects that alter the electronic structure of the material.^18^ In another study, Zhu et al. investigated CO_2_ adsorption on g-C_3_N_4_ and showed that the molecule preferentially binds to two-coordinated nitrogen sites, with moderate adsorption energy (≈−0.42 eV) and clear perturbations to the electronic structure.^19^ Similarly, Liu et al. reported that Pd clusters supported on g-C_3_N_4_ significantly enhance Hg^0^ adsorption, with notable charge transfer and stronger binding compared to the pristine material.^20^ Moreover, Xiong et al. combined experimental and theoretical approaches to confirm the spontaneous adsorption of organic pollutants such as Rhodamine B on g-C_3_N_4_, characterized by favorable adsorption free energies and strong surface affinity.^21^ Collectively, these studies reinforce the versatility of g-C_3_N_4_ and related nanostructures for pollutant capture, supporting its relevance as a candidate material for environmental remediation.

In this study, Grand Canonical Monte Carlo (GCMC) simulations were first employed using the RASPA package to predict the most probable adsorption configurations of 2,6-dichloro-1,4-benzoquinone (DCBQ) on g-C_3_N_4_ monolayers in both triazine (t-g-C_3_N_4_) and tri-s-triazine (h-g-C_3_N_4_) phases. The configurations obtained from these simulations were subsequently refined through first-principles calculations within the DFT framework to determine the structural, energetic, and electronic properties associated with the adsorption process. Noncovalent interaction analyses were then performed based on the electron density and its gradient derived from the DFT calculations, enabling a detailed visualization of weak intermolecular forces through three-dimensional isosurfaces and Reduced Density Gradient (RDG) scatter plots. Additionally, water coadsorption simulations were carried out to mimic realistic aqueous environments. This integrated approach provides new insights into the potential of g-C_3_N_4_ for the selective removal of halobenzoquinones from water and contributes to the advancement of nanostructured materials for environmental remediation.

## 2 Methodology

### 2.1 Structure of g-C_3_N_4_

In this work, two structural forms of g-C_3_N_4_ were considered: the s-triazine ring (C_3_N_3_) and tri-s-triazine ring (C_6_N_7_), which are widely recognized as the fundamental building blocks of this material (see Figure S1 in Supporting Information). The g-C_3_N_4_ is characterized by a stacked two-dimensional lamellar architecture and a π-conjugated polymeric structure, analogous to graphite, with N atoms replacing some C atoms in the graphitic planes linked via sp^2^ hybridization.^22^ This configuration results in dense packing and unique electronic properties. The s-triazine based form consists of smaller cyclic units, while the tri-s-triazine based form features larger units connected via planar tertiary amine.^11^ The choice of these two forms in the present study allows the exploration of relevant structural and electronic differences, contributing to a broader understanding of the adsorption of DCBQ molecules on g-C_3_N_4_ surfaces.

### 2.2 Monte Carlo Simulations

To obtain reliable initial adsorption configurations, Grand Canonical Monte Carlo (GCMC) simulations were carried out using the RASPA program.^23^ This approach enabled us to systematically explore the configurational space of the interactions between 2,6-dichloro-1,4-benzoquinone (DCBQ) molecules and g-C_3_N_4_ surfaces, generating structural arrangements that served as initial geometries for subsequent DFT calculations.

The GCMC simulations were performed under standard conditions, at 298 K and 1 bar. Rigid frameworks were employed for both t-g-C_3_N_4_ and h-g-C_3_N_4_, each modeled using a 1 × 1 × 1 supercell. The t-gC_3_N_4_ system employed lattice parameters *a* = 19.9969 Å, *b* = 12.9116 Å, and *c* = 25.0000 Å, while the h-gC_3_N_4_ system used *a* = 29.5829 Å, *b* = 19.5739 Å, and *c* = 25.0000 Å; in both cases, P1 symmetry with α = β = γ = 90° was adopted.

Each simulation consisted of 20,000 initialization cycles followed by 50,000 production cycles to ensure statistical convergence. The Ewald method was employed to compute long-range electrostatic interactions, with a cutoff distance of 12 Å and a precision of 1 × 10^–4^,^24,25^ while van der Waals forces were treated with the Lennard-Jones potential.^23^ Trial moves included translation, rotation, leave and enter the simulation box, and swap moves with equal probabilities.

The DCBQ molecule was modeled as a rigid adsorbate with all intramolecular bond lengths constrained and no internal degrees of freedom, following the standard RASPA implementation for GCMC simulations.^23^ Nonbonded interactions were described using Lennard–Jones potential, and all parameters used are contained in the RASPA program and are recommended by the program’s authors;^23^ the parameters can be seen in Table S1.

To mimic realistic aqueous environments, additional competitive coadsorption simulations were conducted by systematically varying the molar fractions of DCBQ and water molecules to 50:50, 40:60, 20:80, and 10:90. In these calculations, particle exchange moves ensured equilibrium with external chemical potential, dynamically adjusting the number of molecules during the simulation. Such coadsorption setups are particularly relevant for evaluating the competitive adsorption and selectivity of the DCBQ molecule toward g-C_3_N_4_ in aqueous systems, given that this compound is an emerging waterborne pollutant of high concern. It should be noted that actual aqueous environments contain ions and natural organic matter, which are not included in our simulations. While this simplification may influence predicted adsorption configurations, capacity, and selectivity, coadsorption behavior, and relative selectivity to remain valid, as the dominant interactions (van der Waals and electrostatic forces between DCBQ, water, and the g-C_3_N_4_ framework) are explicitly captured.

The GCMC simulations provided the most favorable adsorption configurations of DCBQ on the g-C_3_N_4_ surfaces, which were subsequently used as initial geometries for DFT-based structural relaxations and electronic property analyses.

### 2.3 Density Functional Theory

In this study, first-principles calculations were based on DFT implemented in the SIESTA package code.^26^ We used the Perdew–Burke–Ernzerhof (PBE) form of the exchange-correlation functional within the generalized gradient approximation (GGA).^27^ The nonlinear exchange-correlation interaction between valence and core electrons is described using Troullier-Martins pseudopotentials.^28^ The valence electron wave functions were represented using a *double*-ζ basis set plus polarization functions (DZP), ensuring an accurate description of electronic interactions, and a mesh *cutoff* of 400 Ry was used in the calculations. The Monkhorst–Pack meshes 5 × 5 × 1 for *k*-point sampling were used for integration over the first Brillouin zone.^29^ The convergence criteria for energy and force were set to 10^–4^ eV and 0.04 eV/Å, respectively.

The g-C_3_N_4_ structures were modeled following the s-triazine model (t-g-C_3_N_4_) and the tri-s-triazine model (h-g-C_3_N_4_), with the DCBQ molecule positioned near the surface to simulate possible interactions. During the calculations, a large 3 × 3 × 1 supercell based on a primitive cell was employed for both structures.

To evaluate the stability of DCBQ molecule adsorption, the adsorption energy (*E*_ads_) was calculated using eq 1:

1 $$E_{ads}=E_{\left(g‐C_{3}N_{4}/DCBQ\right)}-E_{\left(g‐C_{3}N_{4}\right)}-E_{\left(DCBQ\right)}$$

where *E*_(g‑C_3_N_4_/DCBQ)_ represents the total energy of g-C_3_N_4_ (t-g-C_3_N_4_ or h-g-C_3_N_4_) interacting with DCBQ molecule, *E*_(g‑C_3_N_4_)_ represents the total energy of g-C_3_N_4_ without the adsorbed DCBQ molecule, and *E*_(DCBQ)_ represents the energy of the isolated DCBQ molecule.

### 2.4 Noncovalent-Reduced Density Gradient

Non-Covalent Interaction (NCI) calculations were carried out to analyze the nature of the interactions between the systems. The NCI analysis employed the Reduced Density Gradient (RDG) method to identify noncovalent interactions. This method evaluates intermolecular interactions by examining variations in the reduced density gradient (*s*(ρ)) in relation to the electron density (ρ):^30^

2 $$s\left(\rho \right)=\frac{1}{{2\left({3\pi }^{2}\right)}^{1/3}}\frac{\left|\nabla \rho \left(\mathrm{r⃗}\right)\right|}{{\rho \left(\mathrm{r⃗}\right)}^{4/3}}$$

The RDG method specifically identifies noncovalent interactions between atoms by analyzing the electron density distributions (ρ­(*r*)) and gradient behaviors (*s*(ρ)). These are represented in plots where peaks correspond to different interaction types: attractive, dispersive, or repulsive. The generated isosurfaces are color-coded by interaction strength: blue for strongly attractive interactions, such as hydrogen bonds, green for van der Waals interactions, and red for steric repulsions. The NCI maps were constructed using the NCIPLOT software^30^ and visualized with the Visual Molecular Dynamics (VMD) program.^31^

### 2.5 Electronic Sensibility and Recovery Time

The potential application of t-g-C_3_N_4_ and h-*g*-C_3_N_4_ as a sensor for DCBQ also was available in this work. For these analyses, the rate of change in band gap before (*E*_g1_) and after (*E*_g2_) DCBQ adsorption was calculated, due to adsorption affecting the conductivity,^32^ so the signal of current or voltage in the device can be detected.^33^ As a complement data, the eq 3 were used,^34^ which makes to obtain the recovery time (τ),

3 $$\tau ={\nu_{0}}^{-1}e^{{-E}_{b}/k_{b}T}$$

where ν_0_, *K*, and *T* are the attempt frequency, Boltzmann constant, and temperature (298 K), respectively. ν_0_ refers to the frequency of light used to free the electrode surface for reuse; in this case, the following attempt frequencies are used: 1.0 × 10^12^ for infrared light, 5.2 × 10^14^ for yellow light, and 1.0 × 10^16^ for ultraviolet light. Adsorption energy was used as an approximation to the Gibbs free energy variation due to the physical nature of the interaction.^35–37^

## 3 Results and Discussion

### 3.1 2,6-Dichloro-1,4-benzoquinone (DCBQ) Molecule

The DCBQ molecule exhibits a planar and symmetrical geometry, as revealed by the optimized three-dimensional (3D) structure obtained via DFT calculations (see Figure S2a in Supporting Information). This conformation is typical of benzoquinone derivatives, with chlorine atoms occupying the 2 and 6 positions of the aromatic ring. Such spatial arrangement leads to an asymmetric electronic distribution and results in a significant permanent dipole moment, which directly influences the molecule’s reactivity and interaction modes with molecular receptors or surfaces.^38^ A two-dimensional structural representation of DCBQ (see Figure S2b in Supporting Information) further confirms the π-conjugated nature of the system and the location of electronegative atoms along the ring.

To better understand the molecule’s charge distribution, the electrostatic potential (ESP) map was analyzed (see Figure S3 in Supporting Information). This map clearly distinguishes regions of higher and lower electron density. The red areas, concentrated around the oxygen atoms, indicate negative electrostatic potentials—that is, regions of electron accumulation with a higher nucleophilic character. In contrast, the blue areas, associated with the hydrogen atoms and regions adjacent to the chlorines, represent positive potential, meaning electron-deficient zones indicative of electrophilic sites. The green regions correspond to areas of electrostatic neutrality. This asymmetric distribution reinforces the polar nature of the molecule, favoring the occurrence of intermolecular interactions through electrostatic forces and hydrogen bonding, which are relevant in adsorption and molecular recognition processes.^39^

From an electronic perspective, DCBQ displays well-defined the highest occupied molecular orbital (HOMO) and lowest unoccupied molecular orbital (LUMO), with an energy separation of 1.66 eV (see in Supporting Information Figure S4a). This value falls within the typical range for π-conjugated organic molecules and indicates that DCBQ exhibits considerable electronic reactivity, being capable of participating in charge transfer processes.^40,41^ The spatial distribution of the frontier orbitals shows that the HOMO has significant electron density on the chlorine atoms located at positions 2 and 6 of the aromatic ring, as well as on the oxygens from the carbonyl groups (C = O), indicating a doubly localized character. This configuration is associated with electron delocalization arising from the overlap of inductive and resonance effects. In the case of chlorine atoms, although halogens are known for their electron-withdrawing inductive effect (−I), they may also act as electron donors through resonance (+M) when bonded to π-conjugated systems. This occurs due to the ability of their nonbonding electron pairs to interact with the aromatic ring’s π orbitals, allowing a partial contribution of electron density to these peripheral regions.^42,43^

In the case of DCBQ, this resonance effect contributes to the HOMO density observed at the molecular ends, particularly near the chlorine atoms, suggesting that these regions can act as nucleophilic sites in supramolecular (noncovalent) interactions. Additionally, the oxygens of the carbonyl groups also actively participate in the HOMO, mainly through their nonbonding (n) orbitals, which interact with the molecule’s π-conjugated system. This contribution enhances the system’s ability to act as an electron donor, making the carbonyl regions potential interaction sites with electrophilic surfaces. This asymmetric and functionally active HOMO distribution highlights the electronic versatility of DCBQ, whose structure offers multiple nucleophilic sites. This interpretation is consistent with DFT analyses of quinone derivatives, which revealed that HOMO density is localized on carbonyl oxygen and chlorine atoms, confirming the presence of multiple nucleophilic regions in these systems.^44,45^ On the other hand, the LUMO is primarily concentrated on the carbonyl groups, especially over the oxygen and carbon atoms involved in the double bond. The concentration of the LUMO density on these groups characterizes DCBQ as an electron acceptor, favoring interactions with charge-donating surfaces. The overlap of the HOMO on chlorine and oxygen atoms, and the LUMO on the carbonyl groups, reveals an asymmetric electron density distribution that gives DCBQ highly polar and electronically active characteristics. This allows the molecule to act flexibly as both an electron donor and acceptor, facilitating interactions with two-dimensional (2D) semiconductor materials such as g-C_3_N_4_. The polar and electronically active nature of DCBQ favors charge-transfer and adsorption interactions with receptor surfaces like g-C_3_N_4_, a feature widely investigated for applications in aquatic pollutant removal.^46^

Complementing the orbital analysis, the density of states (DOS) profile (see Figure S4b in Supporting Information) provides further insight into the electronic structure of the molecule. The strongly bound states, represented by peaks at lower energy regions, are associated with σ orbitals and the fundamental structure of the aromatic ring. Peaks near the HOMO–LUMO energy separation region correspond to π and nonbonding orbitals, especially from oxygen and chlorine atoms, which corroborates the previous analysis of the frontier orbitals. In summary, the DOS analysis reinforces DCBQ’s electronic profile as a polarized and reactive molecule, indicating the presence of energetically accessible states favorable for electronic interactions with adsorptive surfaces.

### 3.2 Optimized Structure of Monolayer g-C_3_N_4_

The Figure 1a,b show the atomic arrangement of each unit cell of the optimized t-g-C_3_N_4_ and h-g-C_3_N_4_ structures, respectively, revealing C–N bond lengths ranging from 1.35 to 1.46 Å. These values are consistent with the expected sp^2^ hybridization in conjugated polymer networks, confirming the structural stability of both configurations at the atomic level.^47,48^

**Figure 1:**
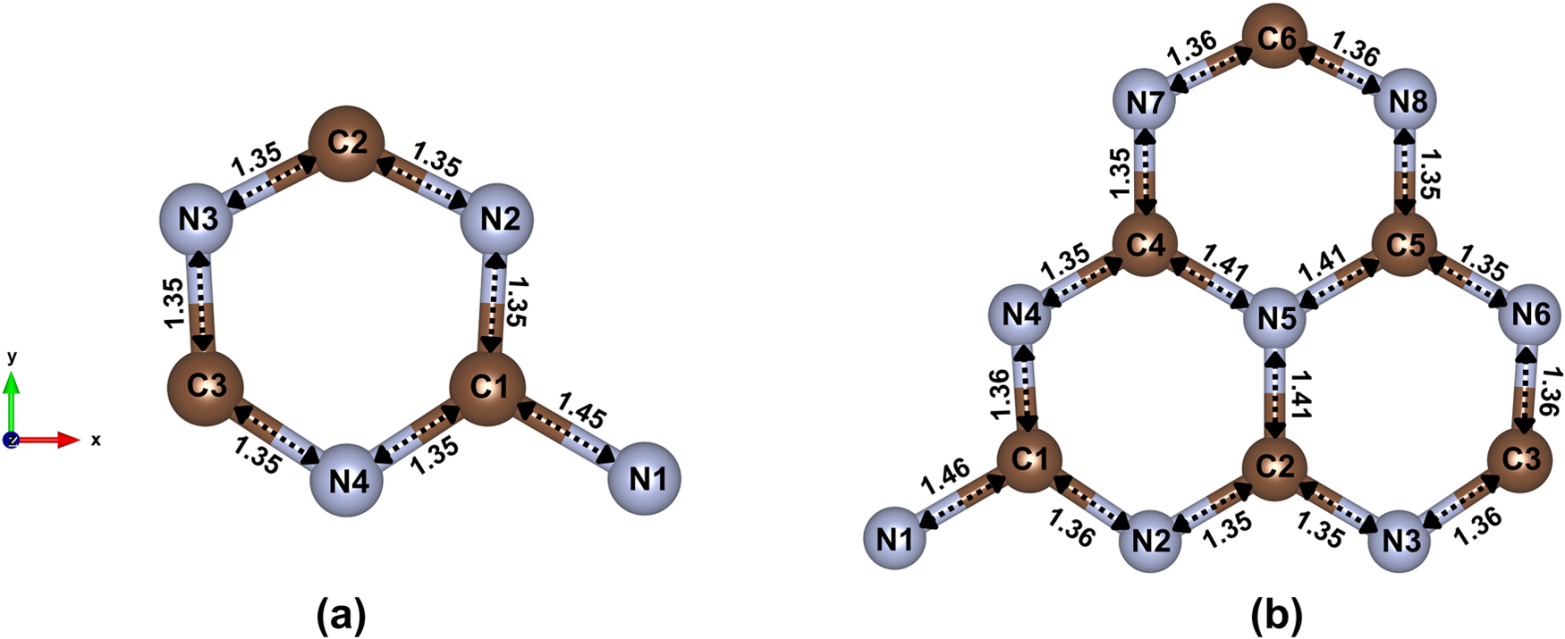
Unit cells of (a) t-g-C_3_N_4_ and (b) h-g-C_3_N_4_ after optimization. C–N bond lengths are given in Å.

To simulate realistic surfaces and allow sufficient space for adsorption studies, each unit cell was replicated into a 3 × 3 × 1 supercell, resulting in total compositions of 63 atoms for t-g-C_3_N_4_ and 126 atoms for h-g-C_3_N_4_. Before geometry optimization, both supercells exhibited perfectly planar surfaces. However, postoptimization, out-of-plane corrugations (wrinkling) appeared (see Figure 2). Such deformation is consistent with observations in other 2D materials where internal strain and flexibility induce wrinkles that affect surface reactivity and local electronic structure. This wrinkling effect, also present to a lesser extent in the t-g-C_3_N_4_ surface, is likely related to internal stress redistribution and flexibility of the π-conjugated framework. Such distortions are typical in large 2D organic systems and can significantly impact the spatial distribution of electrostatic potential and, consequently, the adsorption behavior of external species.^49^

**Figure 2:**
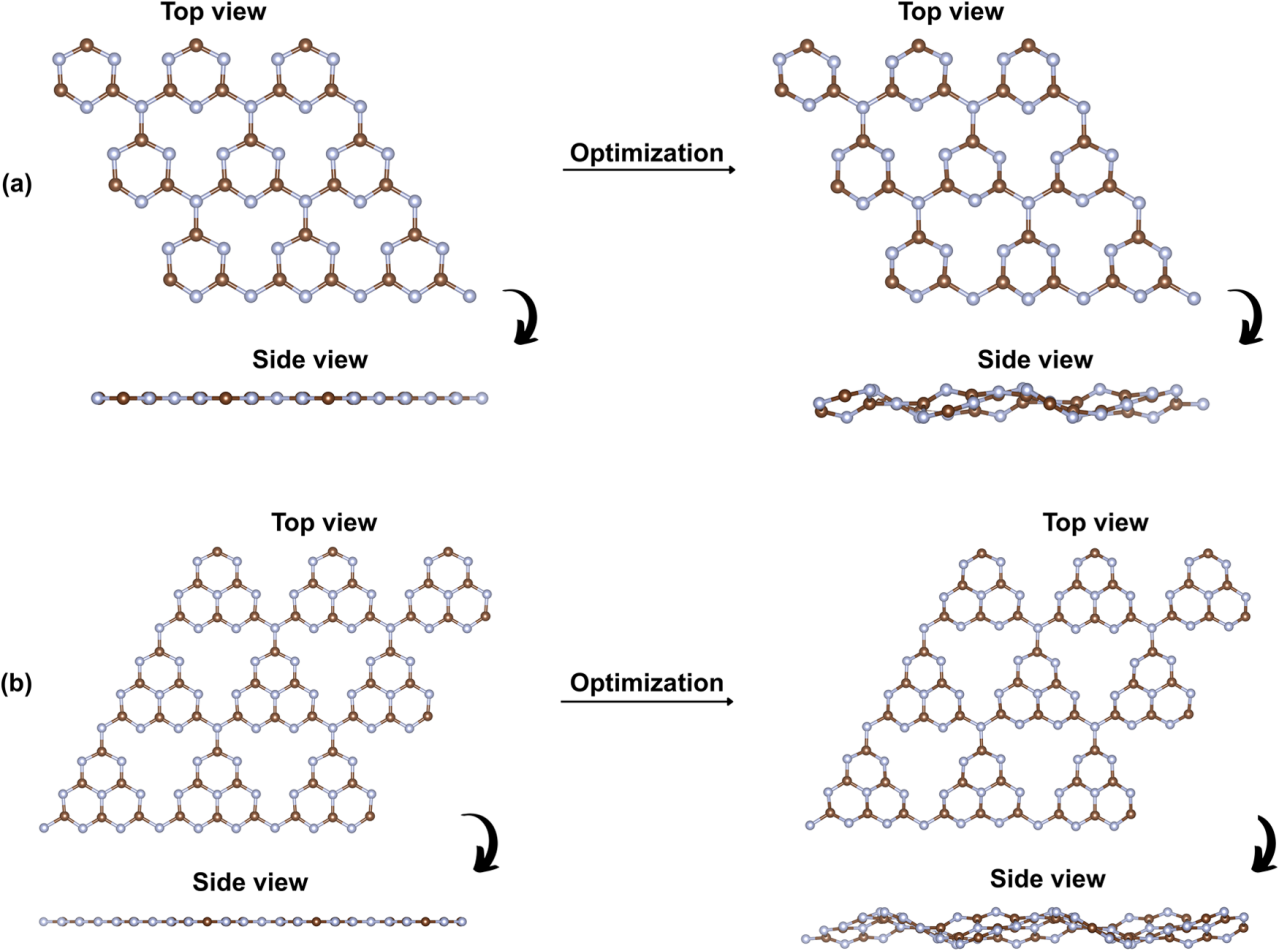
Supercell of (a) t-g-C_3_N_4_ and (b) h-g-C_3_N_4_ before and after structural optimization, showing slight corrugations after relaxation.

To better visualize and understand the electronic surface features of these wrinkled structures, ESP maps were generated, as shown in Figure 3. In both systems, the potential is unevenly distributed: electron-rich regions (negative potential) represented by the green color in Figure 3a–b are concentrated around the nitrogen atoms, particularly at the pore edges, while electron-deficient regions (positive potential) represented by the orange color in Figure 3a–b are located close to the carbon atoms. We infer that the structural undulations introduced during optimization appear to enhance the localization of these electronic regions, potentially creating preferred sites for molecular adsorption, especially in regions of high curvature or charge concentration.

**Figure 3:**
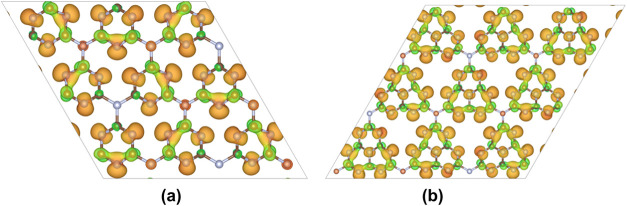
Electrostatic potential (ESP) maps of optimized (a) t-g-C_3_N_4_ and (b) h-g-C_3_N_4_ surfaces, highlighting electron-rich regions (green) and electron-deficient regions (orange).

In summary, the results indicate that both g-C_3_N_4_ configurations provide chemically distinct and electronically active surfaces, with structural and electrostatic characteristics that are likely to affect their interaction with adsorbates such as the DCBQ molecule.

### 3.3 Electronic Properties of the g-C_3_N_4_ and DCBQ Adsorption Effects

#### 3.3.1 Triazine-Based g-C_3_N_4_ (t-g-C_3_N_4_)

The interaction between the t-g-C_3_N_4_ surface and the DCBQ molecule was explored through structural modeling of three different adsorption configurations, as illustrated in Figure 4 showing the initial configurations and the relaxed geometries after complete structural optimization. Following the optimization, the DCBQ molecule remains adsorbed on the surface in all cases but induces local distortions in the t-g-C_3_N_4_ monolayer, including out-of-plane deformations and subtle bending of the molecular structure. These changes are more pronounced when the molecule interacts closely with nitrogen-rich regions, suggesting stronger orbital overlap through π–π stacking and possible charge redistribution. The molecule’s position relative to the π-conjugated system of the triazine framework plays in modulating the interaction strength and, consequently, the electronic response of the material. Theoretical investigations corroborate such surface flexibility and local deformation upon molecular adsorption. Azofra et al. demonstrated that corrugation in g-C_3_N_4_ reduces repulsion between nitrogen lone pairs, stabilizing the Fermi level and enhancing the depth of π-holes, which serve as active sites for CO_2_ conversion.^50^ Additionally, Zhu et al. showed that CO_2_ preferentially adsorbs on two-coordinated nitrogen atoms of g-C_3_N_4_, inducing moderate corrugation of the surface and reinforcing its role as an electron-rich and flexible substrate for adsorption.^19^

**Figure 4:**
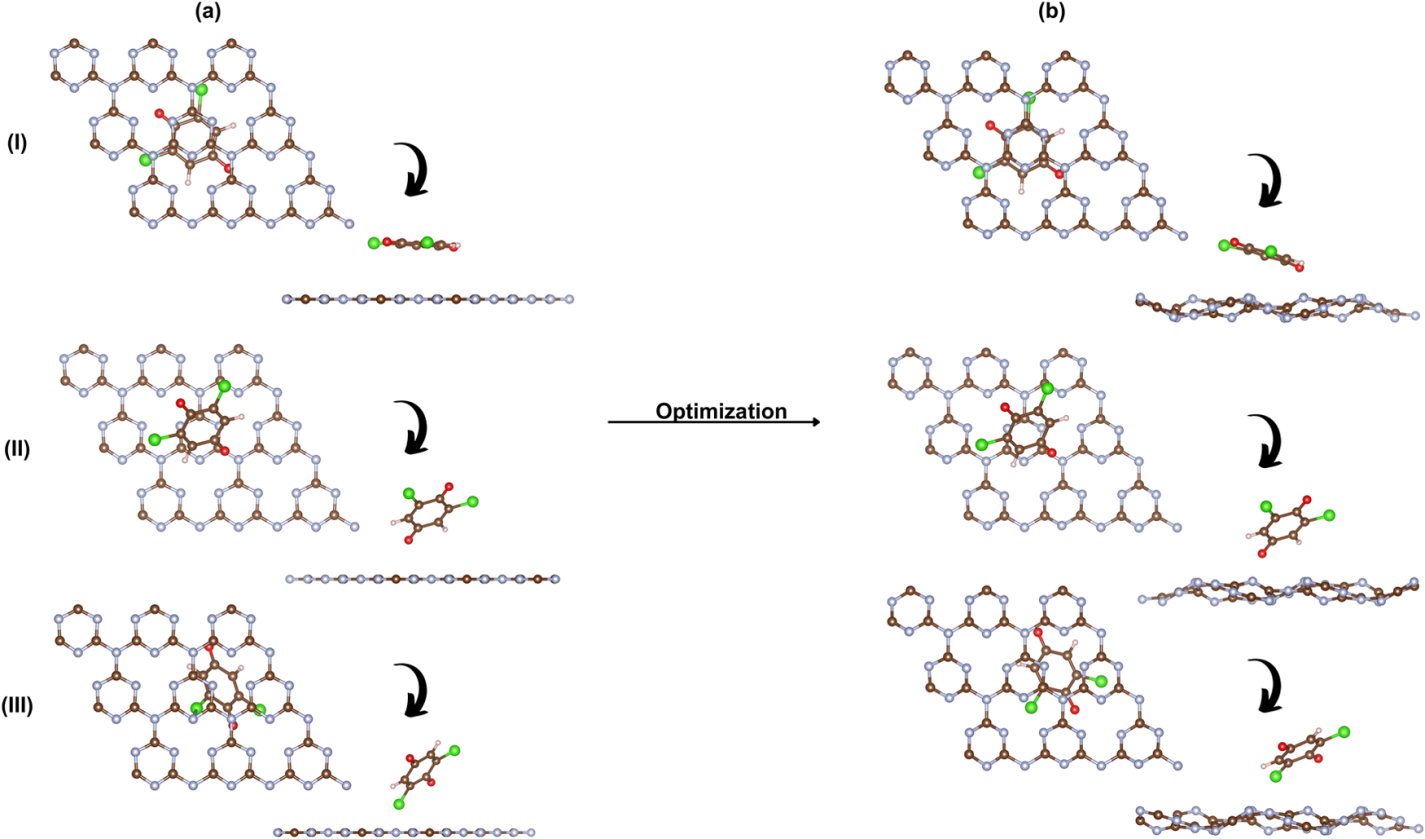
Adsorption configurations of DCBQ on t-g-C_3_N_4_: (a) initial and (b) relaxed structures after optimization for configurations (I)–(III).

The electronic structure of the pristine triazine-based g-C_3_N_4_ (t-g-C_3_N_4_) was first analyzed through band structure and projected density of states (PDOS), as shown in Figure 5a. The system exhibits semiconducting behavior, with a calculated band gap of 2.26 eV, defined between the valence band maximum (VBM) and the conduction band minimum (CBM). This value is consistent with the strong polymorph- and geometry-dependence reported for triazine-based g-C_3_N_4_. Recent ground-state studies indicate that triazine phases frequently relax to buckled (corrugated) geometries, and that buckling can widen the gap by deepening the valence band due to reorientation of N lone pairs. Accordingly, the band gaps among low-energy g-C_3_N_4_ phases can vary substantially depending on the triazine connectivity and buckling degree.^51^ The PDOS reveals that the valence band is predominantly composed of Nitrogen 2p orbitals, while the conduction band is mainly derived from Carbon 2p states as shown in Figure S5 Supporting Information, which is typical of π-conjugated polymeric frameworks.^52,53^

**Figure 5:**
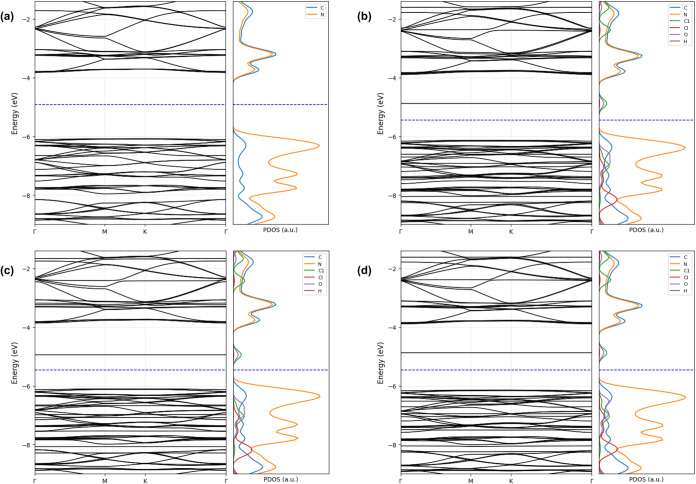
Band structures and PDOS (a) pristine t-g-C_3_N_4_ and (b)–(d) for configurations of the DCBQ molecule adsorbed on the t-g-C_3_N_4_ surface, corresponding to the geometries shown in Figure 4. The dashed blue line indicates the Fermi level.

Upon adsorption of the DCBQ molecule, significant modifications are observed in both the band structure and the PDOS, as illustrated in Figure 5b–d. In the three cases, electronic structure is perturbed, with the appearance of new states located near the Fermi level and a notable reduction in the band gap, indicating electronic coupling between the molecule and the surface. In Figure 5b, corresponding to configuration (I) in Figure 4, DCBQ adsorption induces a reduction in the band gap to 1.25 eV, accompanied by the appearance of new states near the valence and conduction band edges. The PDOS analysis reveals significant contributions from the Chlorine and Oxygen atoms of the DCBQ molecule around the Fermi level, indicating orbital hybridization with the g-C_3_N_4_ surface. This configuration exhibits the strongest interaction, with an adsorption energy of −0.43 eV and a Mülliken charge transfer of +0.048 e^–^, suggesting a stable and electronically active interface. For the system shown in Figure 5c, corresponding to configuration (II) in Figure 4, a more pronounced narrowing of the band gap is observed, reaching 1.16 eV, the lowest value among the three configurations.

The DCBQ-induced states are more distinct within the midgap region, indicating increased electronic coupling with the substrate. Despite the moderate adsorption energy of −0.29 eV and a lower charge transfer of +0.022 e^–^. In contrast, the configuration (III) of Figure 4 maintains a band gap of 1.27 eV, closer to that of the pure system (see Figure 5d). The PDOS shows less pronounced DCBQ states, suggesting weaker orbital overlap. This case is associated with the lowest adsorption energy of −0.24 eV and a modest charge transfer of +0.026 e^–^, indicating limited interaction between the molecule and the surface.

#### 3.3.2 Tri-s-Triazine-Based g-C_3_N_4_ (h-g-C_3_N_4_)

To investigate the influence of DCBQ adsorption on the electronic structure of h-g-C_3_N_4_, three distinct configurations were evaluated (see Figure 6). After structural relaxation, all configurations exhibit noticeable out-of-plane deformations of the surface, more pronounced than those observed in t-g-C_3_N_4_. These distortions are concentrated around the adsorption sites, particularly within the tri-s-triazine pores, which accommodate molecular rearrangement. This flexibility likely facilitates electronic coupling, as corroborated by changes in the electronic structure.

**Figure 6:**
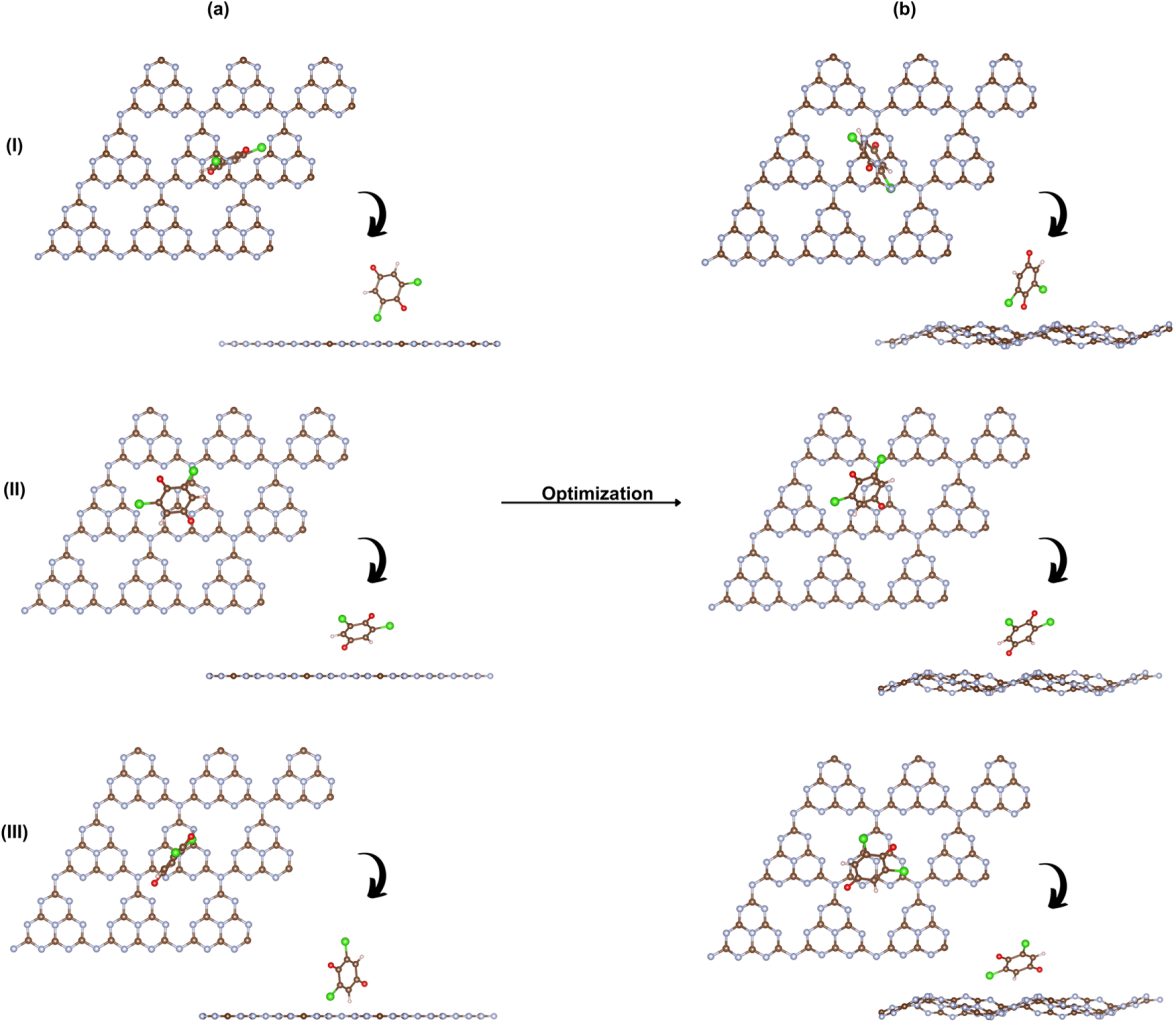
Adsorption configurations of DCBQ on h-g-C_3_N_4_: (a) initial and (b) relaxed structures after optimization for configurations (I)–(III).

The pristine h-g-C_3_N_4_ monolayer exhibits semiconducting behavior with a band gap of 1.78 eV (Figure 7). This value is consistent with previous first-principles studies on tri-s-triazine based g-C_3_N_4_ employing GGA-PBE exchange–correlation functionals, which typically report band gaps in the range of approximately 1.60–2.00 eV for this phase.^54^ In contrast, experimental optical measurements report a larger band gap of around 2.7 eV.^55,56^ Similar to the triazine-based structure, the projected density of states (PDOS) indicates that the valence band maximum is mainly composed of Nitrogen 2p orbitals, whereas the conduction band minimum is primarily associated with Carbon 2p states, as shown Figure S6 of Supporting Information. This orbital arrangement reflects the typical π-conjugated character of g-C_3_N_4_ networks, with tri-s-triazine units offering a more porous and flexible framework.^52,53^

**Figure 7:**
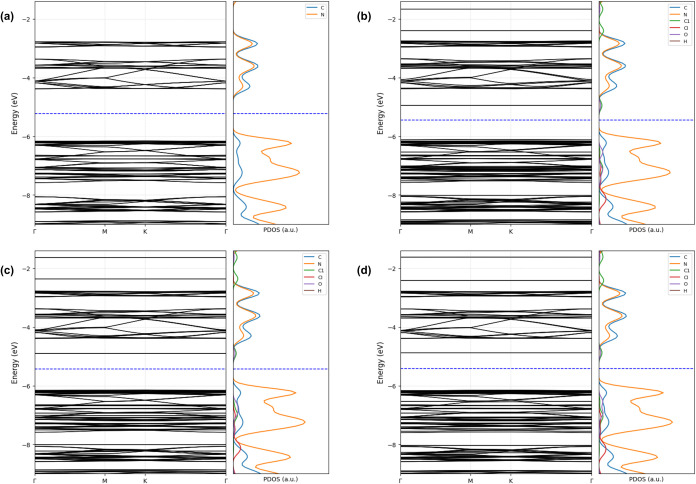
Band structures and PDOS (a) pristine h-g-C_3_N_4_ (b)–(d) for configurations of the DCBQ molecule adsorbed on the h-g-C_3_N_4_ surface, corresponding to the geometries shown in Figure 6. The dashed blue line corresponds to the Fermi level.

The corresponding band structures and PDOS for the three adsorption configurations are presented in Figure 7. Each configuration produces distinct modifications to the electronic profile of the h-g-C_3_N_4_ surface, depending on the interaction strength and charge redistribution. In Figure 7a, corresponding to configuration (I) in Figure 6, the molecule exhibits the strongest interaction with the surface, characterized by an adsorption energy of −0.39 eV and a charge transfer of −0.018 e^–^, suggesting that DCBQ acts as a mild electron acceptor. This interaction leads to a band gap reduction to 1.16 eV, and the PDOS reveals clear contributions from Chlorine and Oxygen orbitals near the Fermi level, evidencing orbital hybridization with the substrate. For the system shown in Figure 7b, which corresponds to configuration (II) in Figure 6, the interaction is weaker, with an adsorption energy of −0.26 eV and a charge transfer of +0.012 e^–^, implying that DCBQ behaves here as a slight electron donor. The band gap is 1.25 eV, and although the molecular states introduced by DCBQ are less pronounced than those in configuration (I), they still affect the band edge profile. Finally, in Figure 7c, corresponding to configuration (III) in Figure 6, represents the weakest interaction among the three systems, with an adsorption energy of −0.23 eV and the same charge transfer of +0.012 e^–^. The band gap is reduced to 1.28 eV, and the PDOS shows subtle hybridization effects.

The band gap modulation observed for both t-g-C_3_N_4_ and h-g-C_3_N_4_ upon DCBQ adsorption is consistent with previous DFT-based studies on semiconductor systems, where interfacial electronic effects have been shown to influence the density of states near the Fermi level and charge distribution. For example, S-scheme heterojunctions such as CdS/La–Bi_2_WO_6_ demonstrate that electronic redistribution at interfaces can significantly alter the electronic response of the material.^57^ In contrast, in the present case, the interaction is predominantly governed by physisorption, and although minor electronic perturbations are detected, no substantial modification of the intrinsic conductivity of the g-C_3_N_4_ phases is observed.

### 3.4 Noncovalent-Reduced Density Gradient Analysis

To further elucidate the nature of the interactions between the DCBQ molecule and the g-C_3_N_4_ monolayers, NCI analysis was carried out using the RDG approach. This method allows the identification and characterization of weak interactions—such as van der Waals forces, hydrogen bonding, and steric repulsion—based on the electron density and its derivatives. The generated 3D isosurfaces and 2D RDG scatter plots provide valuable insight into the strength and spatial distribution of these intermolecular interactions.

For the t-g-C_3_N_4_, as shown in Figure S7a–c of Supporting Information, all analyzed configurations exhibit green isosurfaces between the DCBQ molecule and the material’s surface, which are particularly visible between the Oxygen and Chlorine atoms of DCBQ and the N atoms of the g-C_3_N_4_ sheet, and indicate the predominance of van der Waals forces. This type of interaction is typical of physisorption processes and confirms the absence of significant covalent bonding contribution. The corresponding RDG scatter plots display peaks near zero on the sign­(λ_2_)­ρ axis, further supporting the dominance of weak attractive forces. No blue isosurfaces or significant peaks in the negative region of the RDG plots are observed, suggesting the absence of strong hydrogen bonding or other electrostatic interaction. Likewise, the minimal presence of red isosurfaces, generated mainly through intramolecular interactions within the aromatic ring, supports a scenario with negligible intermolecular steric repulsion, reinforcing that dispersive noncovalent interactions primarily govern the adsorption mechanism.

For h-g-C_3_N_4_, whose results are presented in Figure S8a–c of Supporting Information, green isosurfaces also dominate the interaction regions between DCBQ and the surface, indicating similar van der Waals-type interactions. These isosurfaces appear slightly more spatially extended, likely due to the more open and flexible structure of the heptazine framework, which facilitates physical accommodation of the adsorbate with reduced steric hindrance. In some configurations, such as Figure S8c in Supporting Information, faint blue regions are visible near the oxygen atoms of DCBQ and the N atoms of the surface, suggesting weak electrostatic or dipole–dipole interactions. The RDG scatter plots support these observations, showing high point density in the green region of the sign­(λ_2_)­ρ spectrum and minimal contributions in the red or blue extremes, once again confirming a physisorption-dominated process.

Overall, the NCI/RDG analysis confirms that noncovalent interactions primarily drive the adsorption of DCBQ on both g-C_3_N_4_ phases. No covalent bonding is observed, the dispersive forces and modest charge redistributions are sufficient to induce structural deformation and electronic property modifications in the g-C_3_N_4_ substrates, as previously evidenced in the electronic structure analyses.

### 3.5 Effect of Water Addition to the Systems

To evaluate the influence of water molecules on the adsorption behavior of DCBQ, additional simulations were performed using the RASPA code, incorporating increasing proportions of water in the system. The goal was to simulate more realistic aqueous environments and assess the selectivity and robustness of the g-C_3_N_4_ surfaces toward DCBQ molecule removal under competitive adsorption conditions. The Figure S9, in Supporting Information, illustrates the number of adsorbed DCBQ molecules (in molecules per unit cell) as a function of the water fraction (i.e., the molar percentage of water relative to the total number of molecules) for both triazine-based (t-g-C_3_N_4_) and tri-s-triazine-based (h-g-C_3_N_4_) structures.

It is clearly observed that the h-g-C_3_N_4_ system exhibits a higher adsorption capacity for DCBQ across all analyzed water fractions (50, 60, 80, and 90%), with an average of 43.00 molecules per unit adsorbed. The presence of water has little to no significant impact on this capacity, indicating that h-g-C_3_N_4_ demonstrates high selectivity toward DCBQ even in diluted aqueous environments. In contrast, the t-g-C_3_N_4_ system adsorbed an average of 19.25 molecules per unit, with limited variation in response to the water content, yet showing significantly lower performance compared to h-g-C_3_N_4_. However, the simulation used unit cells with different surface areas: the h-g-C_3_N_4_ area was 1157.80 Å^2^, and the t-g-C_3_N_4_ area was 516.40 Å^2^. Dividing the number of molecules by their respective areas shows that both have approximately the same efficiency, which is 0.037 molecules/Å^2^.

The results suggest that both triazine-based and tri-s-triazine-based structures provide accessible active sites and stronger affinity for DCBQ, even in the presence of water—likely driven by more favorable van der Waals interactions and better accommodation of the molecule. Adsorption in different water proportions generated adsorptions between 3304.5 and 3671.6 mg/g, which is a promising result when compared to the adsorption of 109.3 mg/g obtained by Sun et al. when studying the adsorption of DCBQ on Fe_3_O_4_@SiO_2_@PDA@NH_2_-MIL-53­(Fe).^58^

So, the g-C_3_N_4_ is an efficient structure for DCBQ capture where water is abundant, making it a more promising adsorbent material for applications targeting the removal of organic pollutants from aqueous systems. Simulations with a water content of approximately 99.9999% showed that one molecule of DCBQ is adsorbed in h-g-C_3_N_4_, and it is estimated that a sheet of h-g-C_3_N_4_ with an area of approximately 11.58 μm^2^ can promote the capture of one molecule of DCBQ when concentrations on the order of 10 ng/L are evaluated.

It should be noted that actual water bodies contain ions and natural organic matter, which are not included in our simulations. Moreover, the molar fractions of DCBQ used in the simulations (10–50%) are considerably higher than those typically found in natural water (ng/L range). These elevated concentrations were necessary to ensure adequate sampling of the configurational space in the GCMC simulations, allowing the system to explore multiple adsorption arrangements and cooperative interactions between molecules. While the hydrophobic effect could enhance adsorption at very low DCBQ concentrations, in our system the adsorption is primarily governed by strong molecule–surface and intermolecular interactions. Indeed, even at the lowest simulated DCBQ fraction (10%), the adsorption capacity remains close to that observed at higher fractions, indicating that the dominant interactions between DCBQ molecules and the g-C_3_N_4_ framework, as well as intermolecular stabilizations, control the adsorption behavior.

To further explore the cooperative behavior suggested by GCMC simulations, additional DFT calculations were performed on DCBQ dimers (two molecules interacting in the absence of the substrate). Two representative minima were identified: a local minimum and a global minimum, with adsorption energies of −0.29 and −0.20 eV, respectively. These values indicate that, once an initial DCBQ molecule is adsorbed, additional molecules can stabilize through intermolecular interactions, which explains the cooperative adsorption tendency observed in the GCMC simulations (Figure S9 in Supporting Information). The NCI and RDG analyses provide further insight into these stabilizing mechanisms. For the local minimum (Figure S10a–b), the NCI isosurfaces reveal limited green patches, indicative of localized van der Waals contacts between peripheral atoms, consistent with a less compact geometry. In contrast, the global minimum (Figure S10c–d in Supporting Information) displays more extended green isosurfaces, characteristic of stronger π–π stacking interactions between the aromatic rings. The corresponding RDG plot further supports this interpretation, showing a denser distribution near sign­(λ_2_)­ρ ≈ 0, which is typical of dispersion-dominated stabilization.^50^ The predominance of green regions in the NCI plots confirms the presence of attractive noncovalent interactions, demonstrating that DCBQ molecules not only adsorb strongly on g-C_3_N_4_ but also interact favorably with each other. Together, these results highlight that cooperative molecular packing significantly contributes to the overall adsorption process, enhancing stability and reinforcing the role of g-C_3_N_4_ as a selective adsorbent in aqueous environments.

### 3.6 The g-C_3_N_4_ as a DCBQ Potential Sensor

The calculated band gaps before (*E*_g1_) for both t-g-C_3_N_4_ and h-g-C_3_N_4_ are 2.26 and 1.78 eV respectively, and others available data related to change in band gap to different configurations studied, are listed in Table 1. For t-g-C_3_N_4_, the rate of change in band gap from −43.81% (configuration III) to −48.67% (configuration II), indicating a significant modulation of electronic properties upon adsorption. In contrast, h-g-C_3_N_4_ shows smaller changes, with rate of change in band gap ranging from −28.09 to −34.83%, reflecting lower sensitivity to DCBQ. These results suggest that t-g-C_3_N_4_ exhibits higher electronic sensitivity than h-g-C_3_N_4_, with configurations I and II showing the largest band gap variations.

**Table 1:** Band Gap of g-C_3_N_4_ after DCBQ Adsorption (*E*_g2_) and Rate of Change in Band Gap (Δ*E*_g_/*E*_g1_), for Different Systems Configuration

**configuration**	**g-C**_**3**_ **N**_**4**_	***E***_**g2**_ **(eV)**	**Δ** ***E***_**g**_ **/** ***E***_**g1**_ **(%)**
I	t-C_3_N_4_	1.25	–44.69
	h-C_3_N_4_	1.16	–34.83
II	t-C_3_N_4_	1.16	–48.67
	h-C_3_N_4_	1.25	–29.77
III	t-C_3_N_4_	1.27	–43.81
	h-C_3_N_4_	1.28	–28.09

Recovery times (τ) were calculated using eq 3, considering three different attempt frequencies corresponding to infrared, yellow, and ultraviolet light (ν_0_ = 1.0 × 10^12^, 5.2 × 10^14^, and 1.0 × 10^16^ s^–1^, respectively). The results, summarized in Table 2, indicate that the t-g-C_3_N_4_ sensor can rapidly regenerate after DCBQ adsorption, with τ values in the microsecond to nanosecond range depending on the light source. For instance, configuration I under infrared light yields τ = 1.85 × 10^–5^ s (0.18 μs), which is a value within the recommended range for usual applications, demonstrating fast recovery and suitability for real-time sensing and this makes it possible to use the material as a possible sensor^59^ of DCBQ molecules. These results highlight that the sensor’s recovery can be tuned by selecting the appropriate light frequency, providing flexibility for potential applications.

**Table 2:** Recovery Time Values Calculated Using Three Different Attempt Frequencies at 298 K

		**recovery time (s)**		
**configurations**	**g-C**_**3**_ **N**_**4**_	**infrared light**	**yellow light**	**ultraviolet light**
I	t-C_3_N_4_	1.8 × 10^–5^	3.6 × 10^–8^	1.8 × 10^–9^
	h-C_3_N_4_	3.9 × 10^–6^	7.5 × 10^–9^	3.9 × 10^–10^
II	t-C_3_N_4_	8.0 × 10^–8^	1.5 × 10^–10^	8.0 × 10^–12^
	h-C_3_N_4_	2.5 × 10^–8^	4.8 × 10^–11^	2.5 × 10^–12^
III	t-C_3_N_4_	1.1 × 10^–8^	2.2 × 10^–11^	1.1 × 10^–12^
	h-C_3_N_4_	7.7 × 10^–9^	1.5 × 10^–11^	7.7 × 10^–13^

Overall, t-g-C_3_N_4_, particularly in configurations I and II, presents both significant electronic sensitivity and rapid recovery, making it a promising candidate for DCBQ detection in aqueous environments, whereas h-g-C_3_N_4_ shows moderate sensitivity but could still be suitable depending on the operational requirements.

## 4 Conclusion

This study employed Density Functional Theory (DFT) and Monte Carlo simulations to investigate the interaction mechanisms between 2,6-dichloro-1,4-benzoquinone (DCBQ) and two structural phases of graphitic carbon nitride (g-C_3_N_4_): the triazine-based (t-g-C_3_N_4_) and tri-s-triazine-based (h-g-C_3_N_4_) monolayers. The results demonstrated that both materials exhibit semiconducting behavior and undergo structural and electronic modifications upon DCBQ adsorption, with the effects being more pronounced in the h-g-C_3_N_4_ phase.

The adsorption energy calculations revealed a favorable physisorption process for all configurations, governed mainly by van der Waals interactions. The Mülliken charge analysis indicated a small but measurable charge redistribution between the DCBQ molecule and the surfaces, while the band structure and PDOS analyses confirmed significant gap narrowing after adsorption. Notably, the highest rate of change in band gap (48.67%) and a recovery time compatible with sensor application were observed for the t-g-C_3_N_4_ system, indicating that even weak interactions can substantially perturb the electronic structure of the nanomaterial.

The Noncovalent Interaction (NCI) and Reduced Density Gradient (RDG) analyses reinforced the predominance of dispersive forces and ruled out the presence of strong hydrogen bonding or steric repulsion. These findings align with the physisorption mechanism observed in the energetic and electronic analyses.

Importantly, water adsorption simulations using RASPA revealed that h-g-C_3_N_4_ and h-g-C_3_N_4_ adsorb equivalently across all tested water compositions (from 50 to 90% water). This result indicates that g-C_3_N_4_ not only maintains high selectivity toward DCBQ molecule in diluted aqueous environments but also benefits from its more open and porous structure, which enhances molecular accommodation and interaction.

Taken together, the results confirm the superior adsorption performance and structural adaptability of the g-C_3_N_4_ for DCBQ capture. These properties highlight its potential as a promising adsorbent material for the removal of halobenzoquinones from water, contributing to the development of more efficient and selective strategies for environmental remediation.

## Supplementary Material



## Data Availability

All data and information for the reproduction of these works are available in the text.**Siesta code:** https://siesta.icmab.es/siesta/

## Notes

Studied structures, electronic properties, noncovalent interactions, GCMC simulation results, and a table of the force field parameters (PDF)
