# Supplementary material for: Theoretical Study of the Interaction between Graphitic Carbon Nitride and 2,6-Dichloro-1,4-benzoquinone Pollutants: Environmental Applications
Source: ACS Omega. 2026 May 18;11(21):30731–43. doi: 10.1021/acsomega.5c12928 (PMC13234869; doi:10.1021/acsomega.5c12928)
Supplement: Supplementary file 1 [file ao5c12928_si_001.pdf]

Supporting Information:

Theoretical Study of the Interaction between Graphitic Carbon Nitride and 2,6-Dichloro-1,4-Benzoquinone Pollutants: Environmental Applications

Sara Ruth Ramos Rocha[1,3], Nailton Martins Rodrigues[2], Michael González-Durruthy[3] and Silvete Guerini[1]

[1]*Laboratório de Simulação, Universidade Federal do Maranhão, 65080-805, São Luís - MA, Brasil.*

[2]*Laboratório de Química Computacional, Universidade Federal do Maranhão, 65080-805, São Luís - MA, Brasil.*

[3]*LAQV@REQUIMTE/ Department of Chemistry and Biochemistry, Faculty of Sciences, University of Porto, 4169-007 Porto, Portugal*

Supporting Information:

1. Structural Models of Graphitic Carbon Nitride Schematic of the structural forms of g-C_3N_4: the s-triazine ring (C_3N_3) and the tri-s-triazine ring (C6N7).

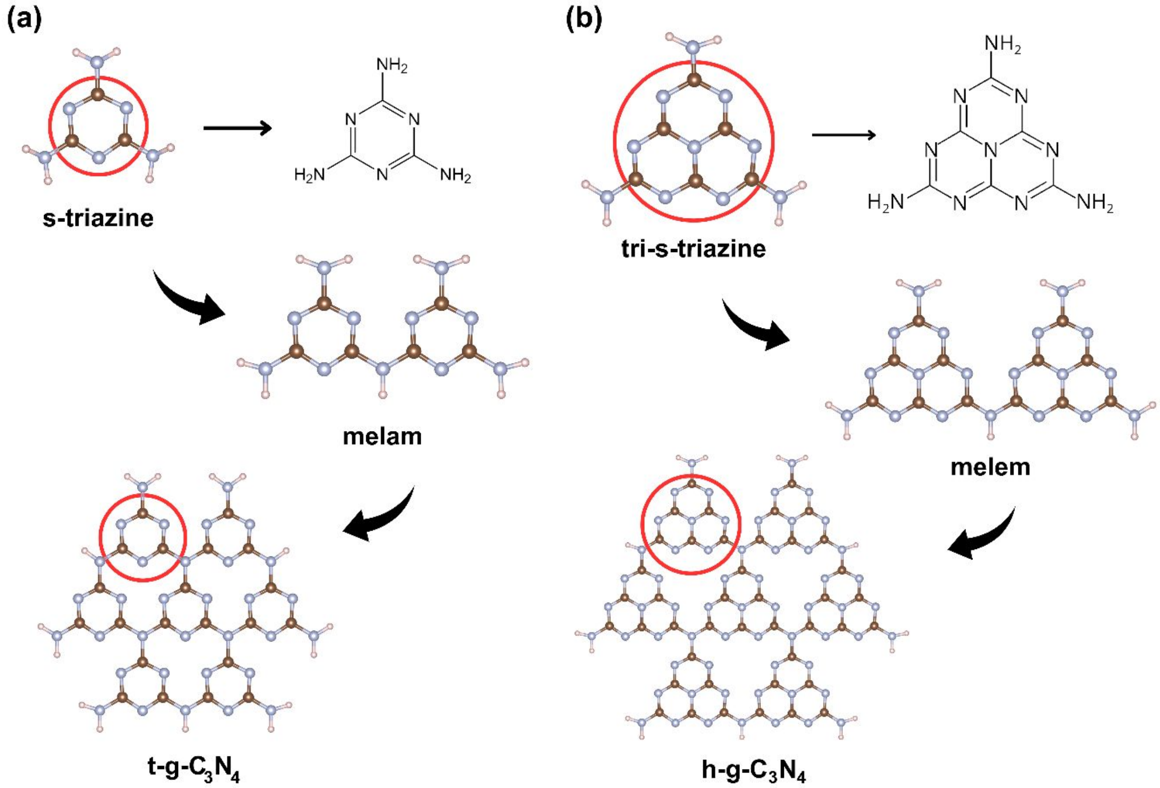

Fig. S1: *The two structures proposed of* g-C_3N_4 *(a)* t-g-C_3N_4 *based on s-triazine and (b) h-*g-C_3N_4

2. Molecular Structure of 2,6-Dichloro-1,4-Benzoquinone

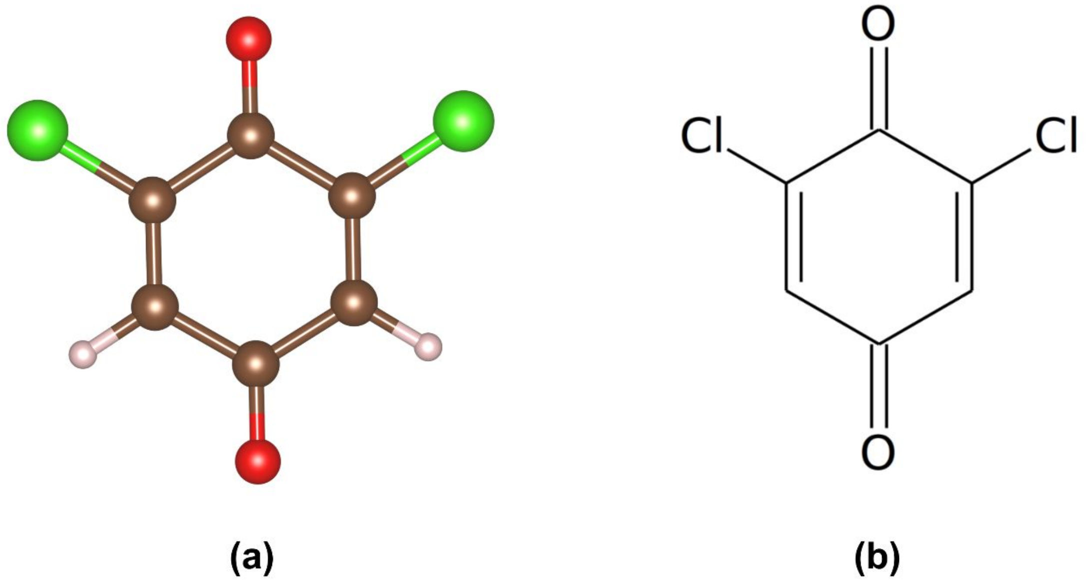

Fig. S2: *Structural representations of 2,6-dichloro-1,4-benzoquinone (DCBQ): (a) optimized 3D molecular geometry obtained by DFT calculations, and (b) 2D structural formula.*

3. Charge distribution map of the 2,6-Dichloro-1,4-Benzoquinone molecule

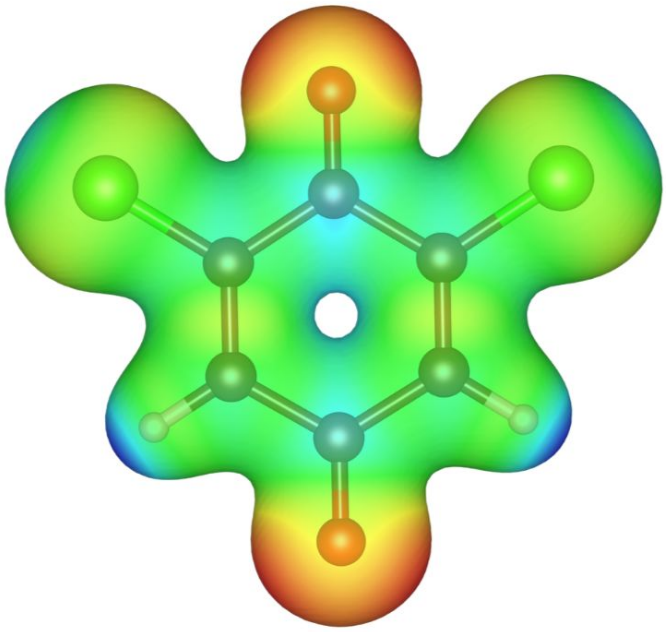

Fig. S3: *Electrostatic potential (ESP) map of the DCBQ molecule.*

4. Electronic properties of the 2,6-Dichloro-1,4-Benzoquinone molecule

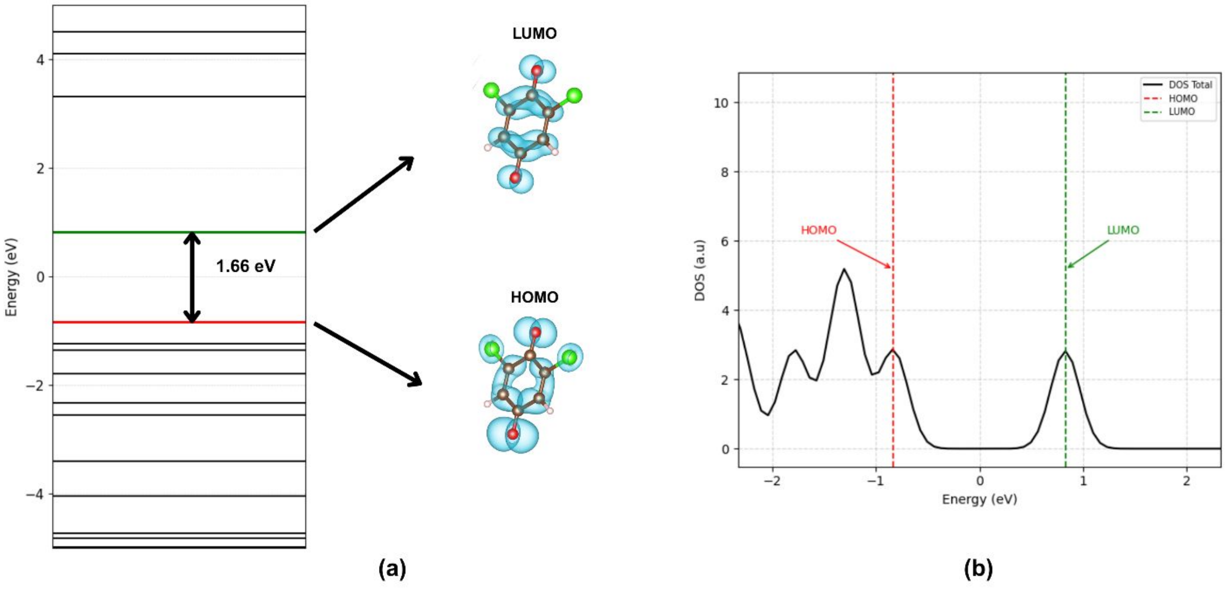

Fig. S4: *Electronic structure of DCBQ: (a) HOMO–LUMO frontier orbitals with 1.66 eV energy separation, and (b) Density of States (DOS) profile.*

5. Representation of the orbitals of the frontier electronic states of g-C_3N_4 Frameworks

To further validate the orbital origin of the frontier electronic states, orbital-resolved PDOS calculations were carried out using the fmpdos module of SIESTA. The results confirm that the valence band edge is primarily composed of N-2p states, while the conduction band edge is dominated by C-2p orbitals for both t-g-C_3N_4 and h-g-C_3N_4, in agreement with the π-conjugated nature of graphitic carbon nitride.

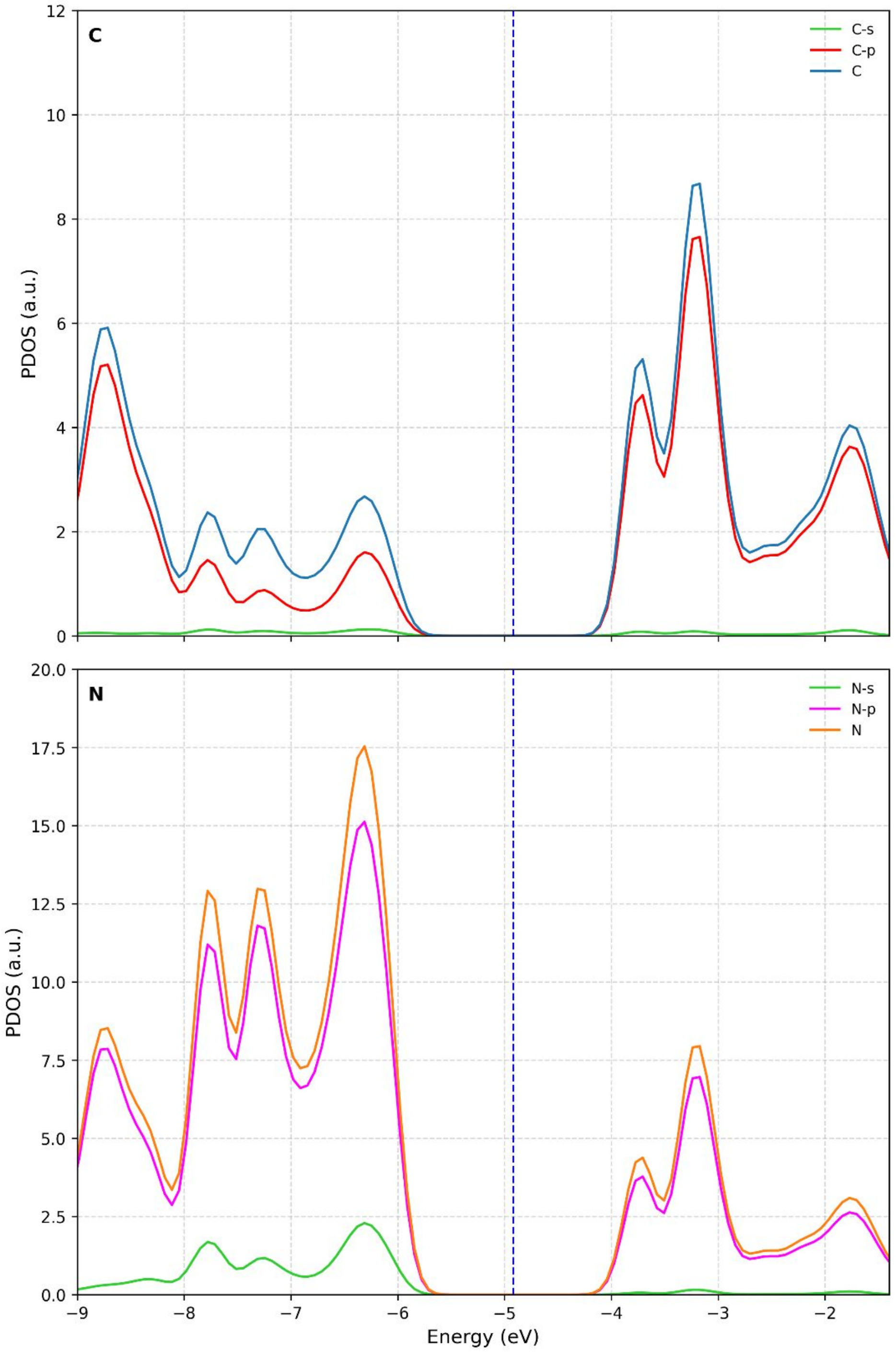

Fig. S5: *Orbital-resolved PDOS of t-g-C_3N_4, decomposed into s and p contributions of carbon (top panel) and nitrogen (bottom panel). The valence band maximum is dominated by N-2p orbitals, while the conduction band minimum is mainly composed of C-2p states, confirming the π-conjugated electronic character of the framework. The vertical dashed line indicates the Fermi level.*

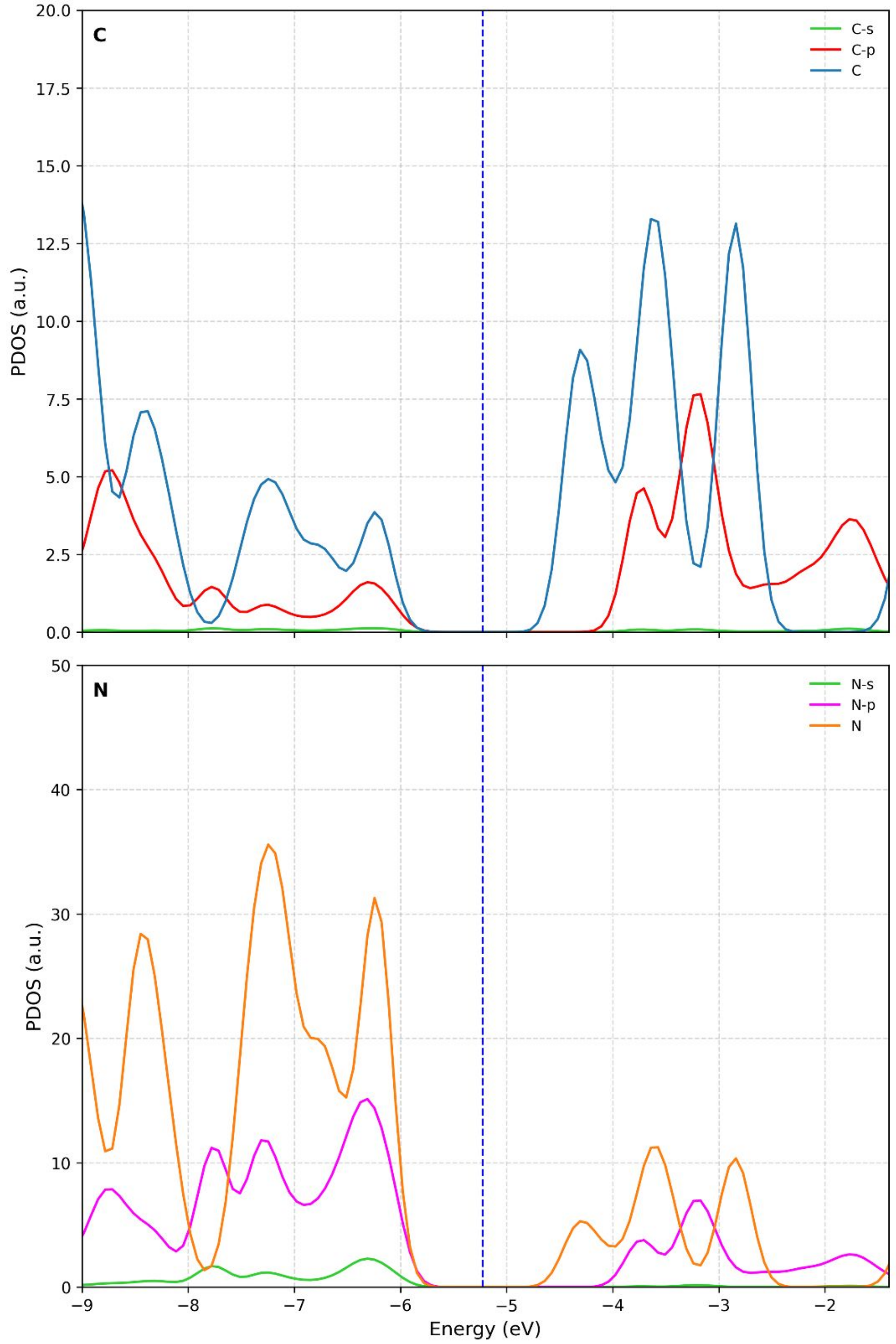

Fig. S6: *Orbital-resolved PDOS of h-g-C_3N_4, showing the s and p orbital contributions of carbon (top panel) and nitrogen (bottom panel). The valence band edge is primarily governed by N-2p states, whereas the conduction band edge is dominated by C-2p orbitals. The dashed vertical line marks the Fermi level.*

6. Noncovalent-Reduced Density Gradient analysis: DCBQ Adsorbed on g-C_3N_4

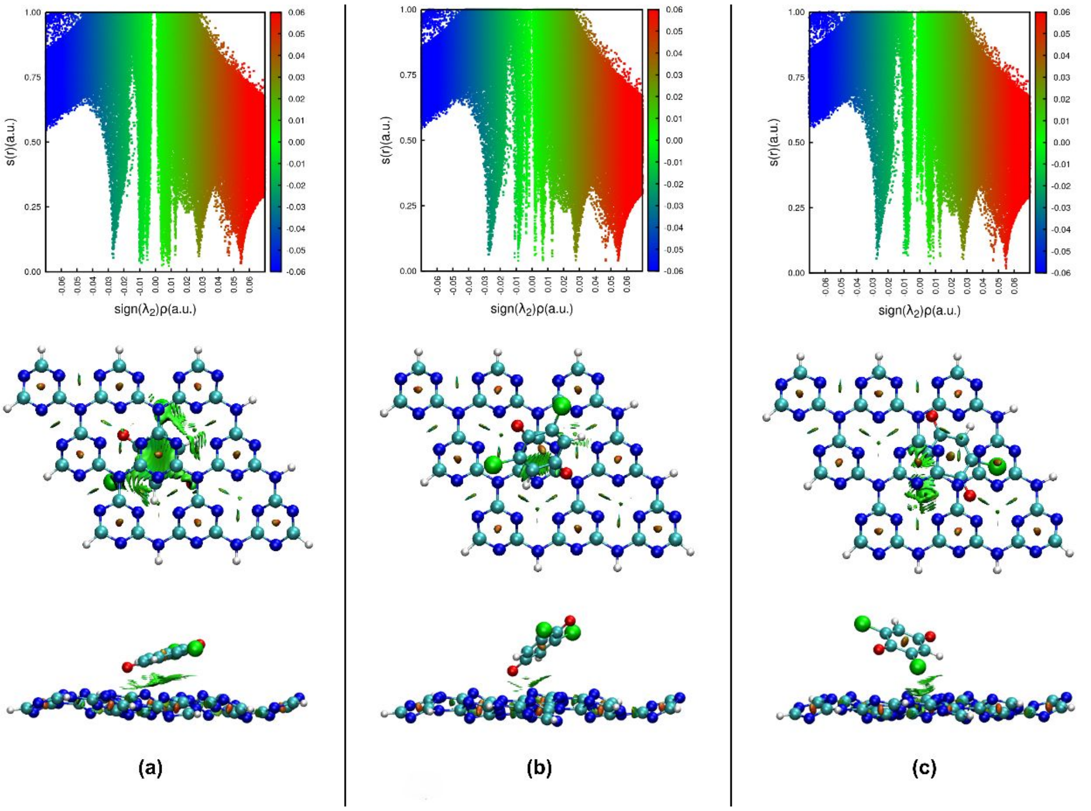

Fig. S7: *NCI isosurfaces and RDG plots for configurations (a)–(c) of the DCBQ molecule adsorbed on the t-g-C_3N_4 surface, corresponding to the adsorption geometries shown in Figure 4.*

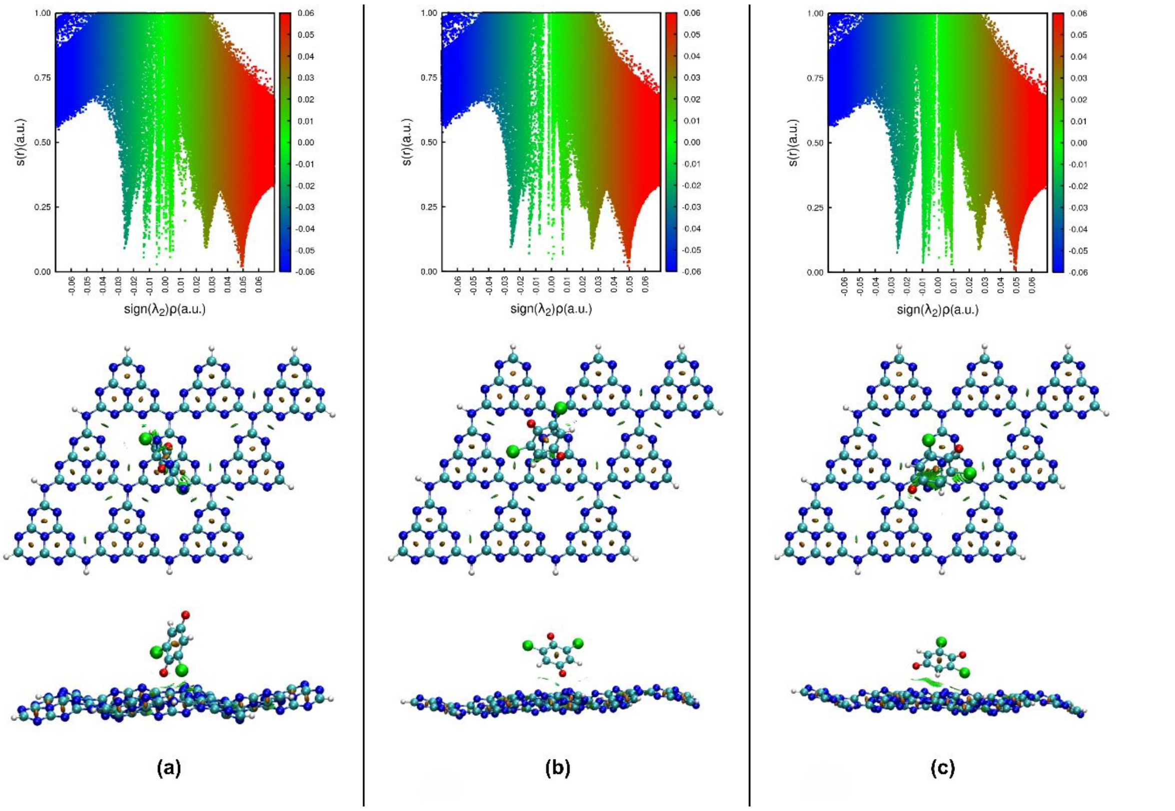

Fig. S8: *NCI isosurfaces and RDG plots for configurations (a)–(c) of the DCBQ molecule adsorbed on the h-g-C_3N_4 surface, corresponding to the adsorption geometries shown in Figure 6.*

7. Effect of water addition on adsorption behavior

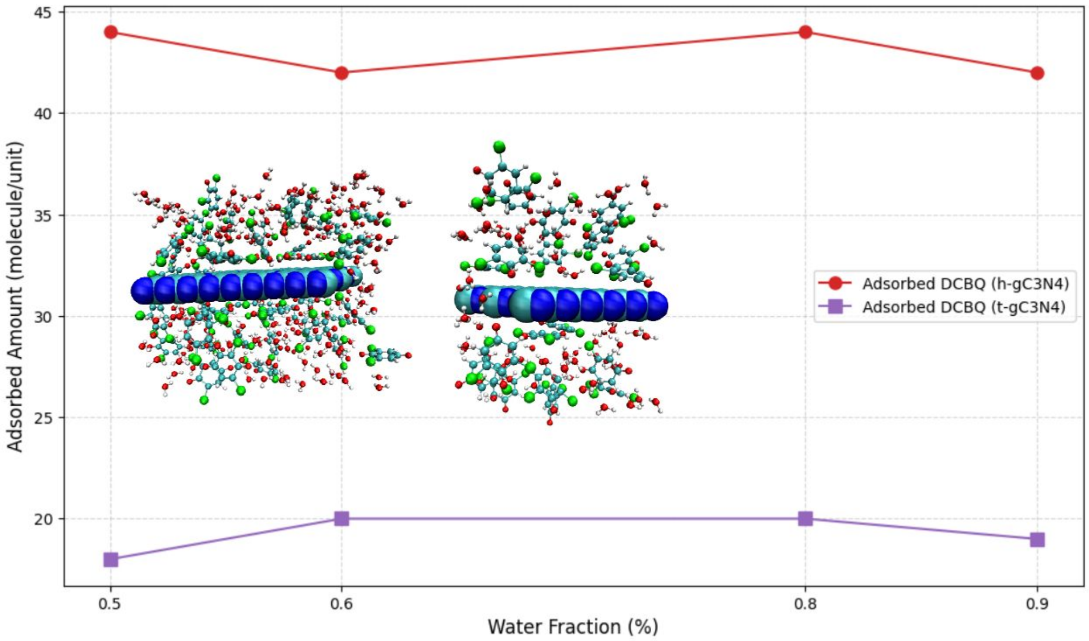

Fig. S9: *Average number of DCBQ molecules adsorbed per unit cell as a function of water fraction for t-g-C_3N_4 and h-g-C_3N_4 systems.*

8. Noncovalent-Reduced Density Gradient analysis: DCBQ Dimers

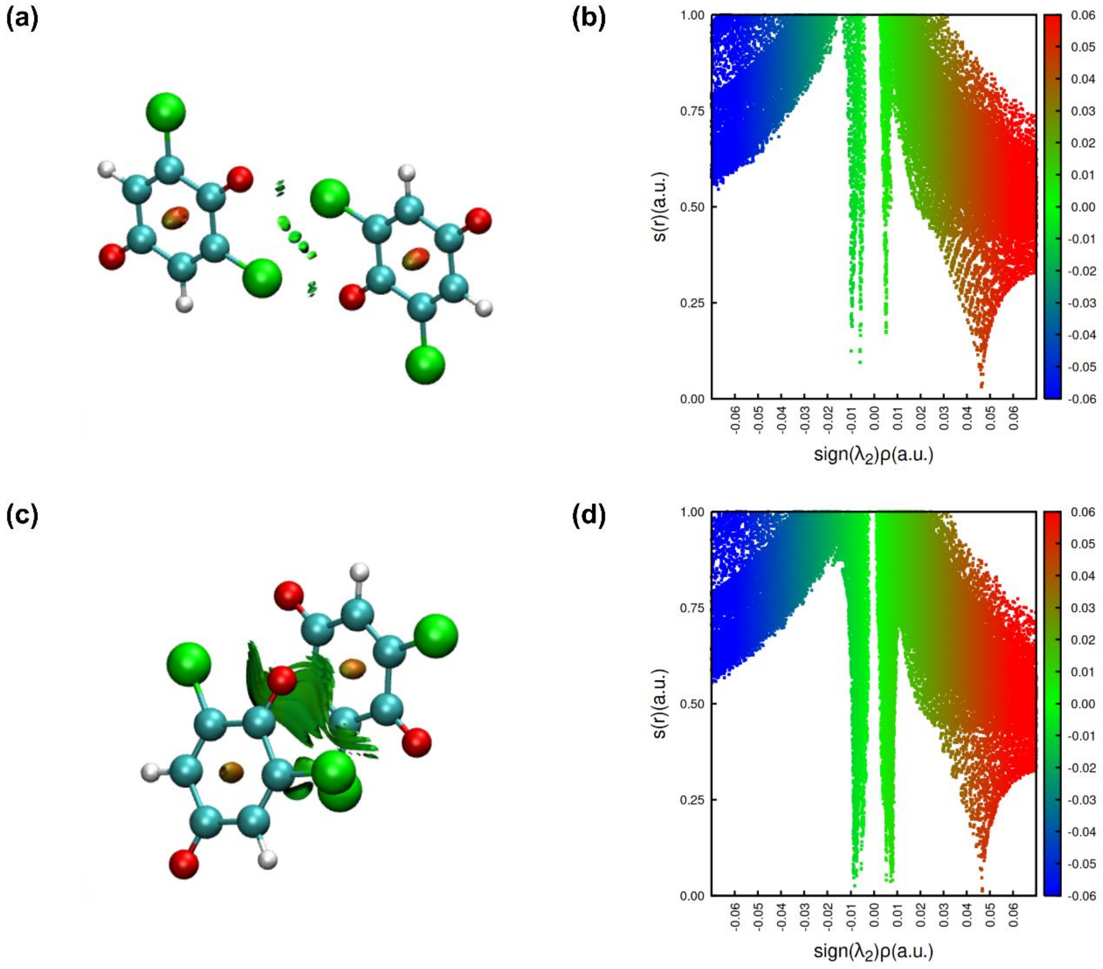

Fig. S10: *NCI isosurfaces and RDG plots for dimers of DCBQ molecules: (a) and (b) local minimum configuration, (c) and (d) global minimum configuration.*

9. *Force Field Parameters*

Table S1. *Lennard–Jones parameters for all atoms of the structures used in the GCMC simulations.*

Structure	***Atom***	***σ (Å)***	***ε/kb (K)***
h-g-C_3N_4	*C*	*3.47299*	*47.8562*
	N	*3.26256*	*38.9492*
DCBQ	*C*	*3.60000*	*30.7000*
	O	*3.03315*	*48.1581*
	Cl	*3.51932*	*142.562*
	H	*2.36000*	*25.4500*
